# Successful thrombolysis with low dose thrombolytic agent in a patient with acute life-threatening massive pulmonary thromboembolism: A case report

**DOI:** 10.1016/j.amsu.2022.104742

**Published:** 2022-09-22

**Authors:** Sagar Adhikari, Nirish Vaidya, Priyanka Poudel, Sujan Pathak

**Affiliations:** a Pulmonary Unit, Department of Internal Medicine, Kathmandu University School of Medical Sciences, Dhulikhel Hospital, Dhulikhel, 45210, Nepal; b Department of Internal Medicine, Kathmandu University School of Medical Sciences, Dhulikhel Hospital, Dhulikhel, 45210, Nepal; c Kathmandu University School of Medical Sciences, Dhulikhel, 45210, Nepal

**Keywords:** Cardiogenic shock, Case reports, Pulmonary thromboembolism, Thrombolytic therapy

## Abstract

**Introduction:**

and importance: Acute massive pulmonary thromboembolism is a potentially life-threatening condition requiring urgent management to decrease mortality. Although the standard dose of systemic thrombolysis with alteplase is 100 mg, half the dose of alteplase can be used to break up clots successfully, especially if bleeding is a concern.

**Case presentation:**

We report a case of massive pulmonary thromboembolism presenting with cardiopulmonary arrest, successfully managed with advanced cardiac life support, anticoagulants, and low-dose thrombolytics.

**Clinical discussion:**

Management of massive pulmonary thromboembolism includes medical thrombolysis along with maintenance of hemodynamic stability. Our patient was successfully managed with low-dose thrombolytics and was continued with standard oral anticoagulants for 6 months.

**Conclusion:**

In patients of acute massive pulmonary thromboembolism, a low dose of the thrombolytic agent can achieve complete resolution of the thrombus with less bleeding risk.

## Highlights

• Acute massive pulmonary thromboembolism is a life-threatening condition requiring urgent diagnosis and management.
• Management of massive pulmonary thromboembolism includes hemodynamic stability and medical thrombolysis.
• Successful thrombolysis can be achieved with low-dose thrombolytics in patients with higher risk of bleeding complications.

## 1 Introduction

Acute Pulmonary Thromboembolism (PTE) is a common cause of cardiovascular death worldwide [1]. The annual incidence rate of PTE is 39–115 per 100,000 people [2]. Since it is a fatal condition and management with thrombolysis has the risk of hemorrhage, clinicians should be aware of emerging treatment modalities for this condition that may improve the chances of success [3]. Here, we report a case of acute bilateral massive pulmonary thromboembolism managed successfully by low-dose thrombolytics.

This case report has been reported in accordance with the SCARE criteria [4].

## 2 Case details

A 40-year-old unconscious female patient was presented to the Emergency Department with a history of central chest discomfort and dyspnea for four days. She did not have any notable risk factors such as immobilization, pregnancy, limb swelling (confirmed negative deep venous thrombosis), hormone replacement therapy, cancer, and previous COVID-19 infection. She has taken COVID-19 vaccine on July 30, 2021 (first dose) and August 29, 2021 (second dose). Her COVID-19 status was negative during admission. She had no hypertension, diabetes mellitus, or other chronic diseases and was not taking any medications before.

**Fig. 1:**
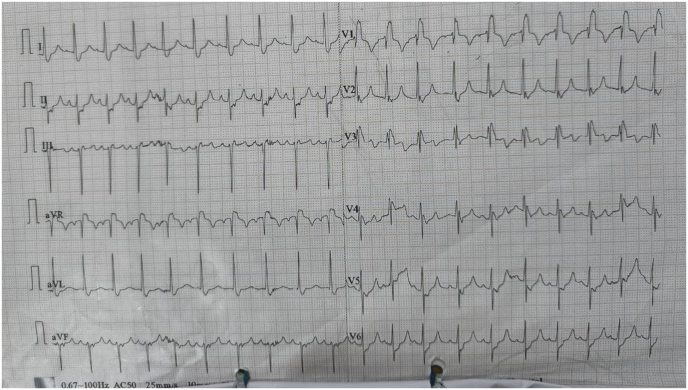
ECG showed Sinus rhythm, right bundle branch block with right ventricular strain pattern.

On clinical evaluation, she was pale and cyanosed. Her Glasgow Coma Scale was 3, hypoxic and the blood pressure couldn't be recorded. Immediate Advanced Cardiac Life Support (ACLS) was started and two cycles of cardiopulmonary resuscitation were given along with endotracheal intubation. There was a return of spontaneous circulation and the patient was shifted to the Intensive Care Unit (ICU) and intravenous vasopressors were given for maintaining blood pressure along with IV fluids. On diagnostic evaluation, electrocardiography (ECG) revealed sinus rhythm and right bundle branch block with right ventricular strain pattern as shown in Fig. 1.

**Fig. 2:**
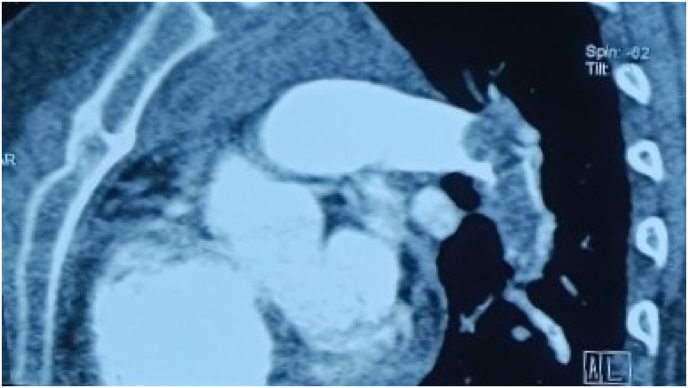
CTPA shows a filling defect in the left pulmonary artery.

The ECG changes were confirmed by bedside echocardiography which revealed right ventricular chamber dilatation along with free wall hypokinesia of dilated right ventricle. Initial diagnosis of acute massive pulmonary thromboembolism (PTE) was suspected and there was a moderate risk score of PTE calculated from Well's score. Subcutaneous Enoxaparin 60 mg was given immediately after confirming the d-Dimer which was 14 μg per milliliter. There was the presence of gross hematuria 2 h following Enoxaparin which resolved spontaneously. Computed Tomography Pulmonary Angiogram (CTPA) showed filling defects in the bilateral main pulmonary artery, and its segmental branches along with subsegmental atelectasis of the posterior segment of the right upper lobe of the lung as shown in Fig. 2. Hence the final diagnosis was confirmed as acute massive pulmonary thromboembolism and planned for intravenous thrombolysis. Enhanced CT was not performed in other parts of the body than the chest.

It was chosen to administer an unusually low dose of thrombolytics to the patient since she experienced hematuria as a result of bleeding issues. Hence, to attempt medical thrombolysis, low dose alteplase 50 mg intravenous was used, followed by unfractionated heparin and six-hourly aPTT monitoring with a target of 60–90 seconds. After 12 hours post thrombolysis with Alteplase, her vasopressors were discontinued. She had improved GCS and arterial blood gas oxygenation with good urine output the next day. As her clinical condition improved over another four days, she was extubated. The patient was shifted to the general medicine ward for further observation the next day. She was clinically asymptomatic the following days and then finally discharged on the tenth day with oral Rivaroxaban.

**Fig. 3:**
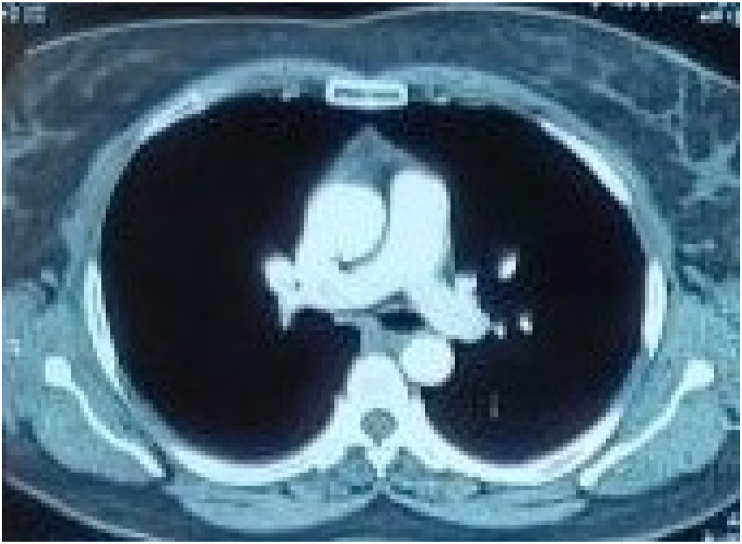
CTPA showed a normal caliber pulmonary artery with no filling defect.

On follow-up after a week, she had no symptoms and the echocardiography was normal. She was continued on oral rivaroxaban. After six months, CTPA was repeated which showed a normal caliber of the bilateral main pulmonary artery with no filling defect in the segmental and subsegmental branches (Fig. 3). Thrombophilia profiles were sent including Protein C, Protein S, Factor V Leiden, Antithrombin III, APLA, and Homocysteine Levels which were normal. Hence, the anticoagulant was stopped. The patient is on regular follow-up and has no symptoms.

## 3 Discussion

Pulmonary thromboembolism (PTE) is the condition where the thrombi originating in deep veins travel into the pulmonary arteries [5]. It is a life-threatening condition that impairs circulation and oxygenation of the blood and can even lead to Right Ventricular (RV) failure; if severe [5]. Major surgery, serious trauma, hormone replacement therapy, increasing age >40 years, malignancy, immobility >3 days, and a previous history of thromboembolic events can result in the formation of venous thrombi which develop emboli that travel to clog the pulmonary arteries of the lungs [2,5].

Occlusion of more than half of the cross-sectional area of the pulmonary arterial system is known as massive pulmonary thromboembolism which is associated with hemodynamic collapse, shock, and right ventricular overload [6]. In our case, the patient presented with chest discomfort and dyspnea along with features of cardiogenic shock.

Hemodynamic instability and RV failure cause early mortality in PTE patients [2]. The mortality rate for massive PTE is 71.4% whereas for low-risk PTE is 28.1% according to a study [7]. More than two third of the deaths in PTE occur within the first hour after the appearance of symptoms necessitating quick assessment and treatment [8]. The patient with massive pulmonary embolism presented with shock, persistent hypotension, and features of RV failure needs immediate cardiopulmonary resuscitation (CPR) followed by volume optimization, vasopressors, inotropes (Noradrenaline/Dobutamine), and mechanical circulatory support (Extra Corporeal Membrane Oxygenation) wherever possible [2]. In our case, we immediately performed two cycles of cardiopulmonary resuscitation along with endotracheal intubation, and intravenous vasopressors were given along with IV fluids.

Clinically, Wells' score is used for the pre-test assessment of PTE [9]. In our patient, there was pulmonary embolism as a more likely diagnosis, and heart rate was more than 100 making the wells' score 4.5 [9]. Wells' score of more than 4 indicates raised pre-test probability of PTE [9]. Furthermore, d-Dimer levels are high in case of acute thrombosis [2]. The d-Dimer value was also elevated in our case strongly suggesting the thromboembolic event. Anticoagulation therapy is initiated in every strongly suspected PTE before further evaluation [10]. As a result of the significant suspicion of continued thromboembolic episodes, we also began enoxaparin subcutaneously. The elevated d-Dimer with high pre-test probability Wells’ score urges further diagnostic imaging for PTE [11]. Here, We performed chest radiography, electrocardiography (ECG), and echocardiography as routine investigations and computed tomography pulmonary angiogram (CTPA) as diagnostic imaging. In our case, the chest radiograph was normal. ECG was suggestive of RV hypertrophy and RV strain pattern. Echocardiography showed free wall hypokinesis of the dilated right ventricle and apical sparing. CTPA showed bilateral main pulmonary artery and segmental branches filling defect confirming the diagnosis of massive PTE with right ventricular failure.

A patient with PTE and cardiogenic shock is managed with a full dosage of the systemic thrombolytic drug, which is 100 mg tissue Plasminogen Activator (tPA) over 2 h, after patient stabilization and anticoagulant medication [2]. In our case, enoxaparin anticoagulation therapy caused gross hematuria after 2 h, thus we chose systemic thrombolysis with an unusual half dosage rather than a full dose of a thrombolytic agent.

Full dose systemic thrombolysis increases the risk of bleeding manifestations and administering a lower dose (50 mg of tPA) in patients having relative contraindications to systemic thrombolysis results in comparable improvements in obstruction, perfusion, pulmonary artery pressure, and right ventricular size with fewer bleeding problems [2]. A study proposed that low-dose thrombolytics are associated with better clinical outcomes in patients with intermediate to high-risk pulmonary embolism [12]. Another study found that in patients with submassive PE, half-dose t-PA reduces hemorrhagic events without causing death or hemodynamic deterioration [3]. Likewise, a case series illustrated low-dose t-PA as a safe treatment option for submassive pulmonary embolism [13]. Here, in our case, an acute massive pulmonary thromboembolism patient, who had returned to spontaneous circulation after CPR, has undergone successful thrombolysis with a non-conventional half dose of a thrombolytic agent which is 50 mg of recombinant tissue plasminogen activator (Alteplase).

Thus, systemic thrombolysis with a half dose of Alteplase is used not only in submassive PTE but also in massive PTE with fewer bleeding complications and also reduces the economic burden on the patient.

## 4 Conclusion

Acute massive pulmonary thromboembolism is a common yet unidentified clinical condition that if left untreated can be life-threatening. However, timely diagnosis and thrombolysis can prevent a significant risk of mortality. The standard dose of systemic thrombolysis with intravenous alteplase is 100 mg. However, successful thrombolysis can also be done with a non-conventional half dose of alteplase in conditions where bleeding risk is a concern.

## Ethical approval

N/A

## Sources of funding

None.

## Authors’ contribution

Dr. Sagar Adhikari and Dr. Nirish Vaidya contributed to clinical management and patient care and manuscript editing.

Sujan Pathak and Priyanka Poudel wrote and edited the manuscript.

## Availability of supporting data

All supporting documents are submitted along with the case report.

## Author contribution

First Sagar Adhikari Clinical management, patient care, manuscript editing.

Second Nirish Vaidya Clinical management, patient care, manuscript editing, Guarantor.

Third Priyanka Poudel Manuscript writing and editing.

Fourth Sujan Pathak Manuscript writing and editing.

## Consent

Written informed consent was obtained from the patient for publication of this case report and accompanying images. A copy of the written consent is available for review by the Editor-in-Chief of this journal on request.

## Registration of research studies

1. Name of the registry: N/A.

2. Unique Identifying number or registration ID: N/A.

3. Hyperlink to your specific registration (must be publicly accessible and will be checked): N/A.

## Guarantor

Dr. Nirish Vaidya, Lecturer, Cardio-pulmonary Unit, Department of Internal Medicine, Dhulikhel HospitalKathmandu University Hospital

## Provenance and peer review

Not commissioned, externally peer reviewed.

## Declaration of competing interest

None.
